# Is it time to move beyond trait self-control?

**DOI:** 10.3389/fpsyg.2024.1435862

**Published:** 2024-08-27

**Authors:** Junhua Dang, Lile Jia

**Affiliations:** ^1^School of Humanities and Social Sciences, Xi'an Jiaotong University, Xi'an, China; ^2^Department of Psychology, National University of Singapore, Singapore, Singapore

**Keywords:** self-control, trait self-control, construct validity, construct redundancy, measurement redundancy

## Introduction

Self-control has been considered as one of the defining features of human nature and defined as the capacity allowing people to override their predominant but maladaptive thoughts, emotions, and behaviors in order to keep them in line with overarching goals (Baumeister et al., 2007). Meanwhile, it has been suggested that there are substantial individual differences existing in people's self-control capacity and the construct of “trait self-control” was developed to indicate such individual differences. The seminal paper proposing this construct and the most frequently used measure of it (i.e., the Self-Control Scale, SCS) has been cited over 9,000 times in Google Scholar and greatly influenced many subdisciplines of psychological sciences (Tangney et al., 2004). Despite their popularity, recent studies call into question the validity of the SCS as well as the scientific usefulness of the construct of trait self-control itself. That is, it is unclear what trait in “trait self-control” refers to and whether “trait self-control” is a redundant psychological construct in our understanding of self-control.

## Validity issues of the SCS

There are at least five validity issues that prevent the SCS from being a good measurement tool. First, although the SCS is designed to measure the ability to inhibit or control undesired behavioral tendencies, many items are unrelated to inhibition (e.g., “I am lazy” or “I have trouble concentrating”). Some researchers argue that there should be two factors included in the SCS, namely “stop control and start control” (de Boer et al., 2011) or “inhibitory self-control and initiatory self-control” (de Ridder et al., 2011a). However, such a two-factor structure is mutable across studies (Ferrari et al., 2009; Maloney et al., 2012; Morean et al., 2014; Hagger et al., 2021) and often shows poorer fit than the original one-factor structure (Lindner et al., 2015; Brevers et al., 2017; Manapat et al., 2021). Note no matter it is inhibitory or initiatory, the SCS aims to measure the efficiency of controlled behaviors. However, meta-analytical evidence suggests that this scale is more strongly associated with automatic behaviors rather than controlled behaviors (de Ridder et al., 2011b).

Second, the SCS targets a general capacity that people can employ to pursue their long-term goals, no matter which domain these goals refer to (e.g., health, academy, and social relationship) and how concrete these goals are (e.g., eating a high-calorie hamburger at this right moment vs. dieting, hanging out before examination vs. getting a master's degree). However, the SCS mixes domain-general items (e.g., “I am good at resisting temptation”) with domain-specific items (e.g., “I spend too much money”), and fails to take goal hierarchy into consideration. Interestingly, the predicting effects of the SCS vary dramatically across life domains (de Ridder et al., 2011b). It is unclear such variation occurs because trait self-control really matters differently in each domain or just because some people value certain goals in specific domains and the SCS happens to include corresponding domain-specific items.

Third, the SCS has a substantial overlap with the personality trait conscientiousness. For example, the SCS includes several items that correspond to the “classic” indicators of conscientiousness (e.g., “I am reliable” and “I am always on time”) (Roberts et al., 2014). Empirically, the correlation between the SCS and the measure of conscientiousness is generally higher than 0.60 and sometime over 0.70 (Costantini and Perugini, 2016; Werner et al., 2019), which means the disattenuated correlation (or the “true correlation,” which equals raw correlation between *x* and *y* divided by the square root of the product of the reliability of *x* and the reliability of *y*) is extremely high (*R* = 0.75–0.86). Even if the SCS is separated into a stop control subscale and a start control subscale, it still has a great overlap with conscientiousness, with an even strong correlation between stop control and the impulse control facet of conscientiousness (*r* =0.77, with an almost perfect disattenuated correlation of *R* = 0.95) as well as a strong correlation between start control between the industriousness facet of conscientiousness (*r* = 0.52, with a disattenuated correlation of *R* = 0.64) (Costantini et al., 2015).

Fourth, people's responses to the SCS suffer greatly from social desirability bias. In the seminal paper, the SCS is substantially associated with measures of social desirability (*r*s = 0.54–0.60) (Tangney et al., 2004). Similarly, a meta-analysis resulting from 81 studies with a total of 24,282 participants also reveals a comparable effect size, *r* = 0.41 (*R* = 0.54 after correcting for attenuation of unreliability) (Zhang et al., 2021). In addition to these cross-sectional findings, longitudinal research shows that the SCS can also prospectively predict follow-up social desirability (even after controlling for baseline social desirability), which means individuals scoring high on the SCS are those who tend to provide socially desirable responses in self-reports (Stavrova and Kokkoris, 2019).

Finally, recent studies reveal that the SCS may even reflect a distorted perception of control mastering. For instance, Jia et al. shows that extreme debtors, whose credit card debt amounts to more than 12 months of their income, report the highest on the SCS but score the lowest on measures of behavioral intention and executive function, which is robust across different subsections of demographics (age, gender, and educational level) and a variety of reasons for indebtedness (Jia et al., 2023). In a similar vein, Astle et al. (2024) examines the relationship between the SCS and the over-claiming questionnaire (OCQ). The OCQ measures perceived familiarity with real and fake information and yields indices of accuracy (i.e., having heard of real items) and bias (i.e., having heard of fake items). Results show that the SCS is negatively correlated with OCQ accuracy but positively correlated with OCQ bias, implying that individuals who score high on the SCS are those who perform worse but claim higher. These findings suggest that in some circumstances the SCS is not at all able to differentiate between individuals who are good at self-control and those who are not. Instead, it measures illusory control over one's life.

## Construct redundance

Not only is the measure of trait self-control problematic, but the construct of trait self-control itself may also be redundant. On the one hand, as a between-person variable, trait self-control is proposed to indicate stable individual differences in self-control capacity, no matter whether it can be captured by the SCS or not. However, recent studies suggest that self-control varies more at the within-person level than at the between-person level. For example, both short-term experience sampling and longitudinal tracking studies show that over 85% of the variance in self-control success (i.e., goal progress) is at the within-person level (Werner et al., 2016; Milyavskaya and Inzlicht, 2017; Roehrick et al., 2023), implying it is not that some people are better at self-control than others. Instead, people are better at self-control in some domains of their lives than in other domains. More importantly, self-control capacity fluctuates greatly from day to day and even more so from moment to moment. For instance, by revising domain-general items of the SCS to measure self-control every evening across three 9-day measurement bursts over 6 months, Schmid and colleagues find < 40% of the variability in daily self-control is attributable to interindividual differences (Schmid et al., 2024). Furthermore, in a study measuring self-control exertion when confronted with a specific temptation in everyday life, only 10% of the total variance is attributable to interindividual differences (Milyavskaya and Inzlicht, 2017).

On the other hand, self-control refers to all means of resolving the conflict between competing goals, typically with a temptation in conflict with a longstanding goal (Inzlicht et al., 2021). Effortfully resisting temptation is only one strategy out of many that people can adopt to resolve conflicts in the self-control dilemma (e.g., situation modification, distraction, and reminding oneself of goals) (Duckworth et al., 2016a, 2018). Specifically, empirical evidence shows that people adopt effortful resistance strategy in only 20% of occasions (Wenzel et al., 2023, 2024). Meanwhile, the effectiveness of effortful resistance is generally weaker than many other strategies (Duckworth et al., 2016b; Williamson and Wilkowski, 2020), often even unrelated to goal attainment (Milyavskaya and Inzlicht, 2017). In contrast, people (including children) tend to employ a variety of strategies (Raghunathan et al., 2023; Wenzel et al., 2023) and prefer different strategies for different temptations (e.g., eating vs. leisure vs. work) (Hennecke et al., 2019; Milyavskaya et al., 2021). They can even intentionally avoid entering the self-control dilemma, which may be more effective in achieving the longstanding goal (Duckworth et al., 2016b). However, there is no one single strategy that is a cure-all. Instead, variability between strategies (Baldwin et al., 2022; Wenzel et al., 2023) and flexibly switching strategies in response to situational demands (Bürgler et al., 2021; Wenzel et al., 2024) are crucial for self-control success. Given the malleability of self-control conflict resolution, it may be difficult to seek an individual difference that differentiates people who are good at self-control from those who are not in all contexts.

## Discussion

Taken together, recent studies have raised doubts about the validity of trait self-control measures and the idea that self-control capacity is a stable individual trait. Although higher scores on measures like the SCS often correlate with positive outcomes, such as improved subjective well-being and better interpersonal relationships, these correlations might be influenced by factors like the overlap between self-control and conscientiousness, as well as social desirability biases. In other words, the positive outcomes associated with high self-control scores might actually reflect shared variance with conscientiousness traits or the tendency of people to present themselves in a socially desirable manner, rather than indicating a true measure of self-control.

The key question we face is which direction we should take. On one hand, if the construct of trait self-control is found to be redundant, we may need to reconsider or abandon it. As an alternative approach, we might consider self-control from a “toolbox” perspective, where self-control success depends on the strategies a person employs, the appropriateness of these strategies for the given context, and how effectively the strategies are implemented (Fujita et al., 2020; Werner and Ford, 2023). Correspondingly, a productive way is to investigate how personal values, personality traits, motivations, and cognitive abilities are related to the selection of self-control strategies and the effectiveness of each strategy (or a combination of several strategies) in a particular domain. For example, people with high conscientiousness often avoid situations and actions that may lead to infidelity, which in turn predicts better romantic relationships (Hill et al., 2014). Openness may be associated with a tendency to flexibly adapt strategy use across different contexts (Wenzel et al., 2024). Those with high working memory capacity may be more competent in handling strategies demanding mental effort (Hofmann et al., 2008).

On the other hand, if the construct of trait self-control is not redundant and stable individual differences in self-control capacity are considered valid, we should continue the validation process to refine and improve its measures. First, we could use domain-general items from the SCS that focus on inhibition to measure trait self-control (Schmid et al., 2024), as these items align more closely with the original definition of the construct (Tangney et al., 2004). Additionally, it is important to re-examine its construct validity, with a particular focus on its discriminant validity, to determine how it differentiates from related constructs like conscientiousness. Second, there may be a higher-order trait of self-control that the SCS only partially captures. For instance, a recent study found that the overlapping features of the SCS, the Short Grit Scale, and the conscientiousness subscale of the Big Five Inventory together predicted academic motivation more effectively than any of these measures individually (Werner et al., 2019). Finally, we could integrate strategy use into the self-control construct and develop a measure that reflects individuals' chronic tendencies in adopting self-control strategies across various typical situations, similar to the approach used in the Emotional Regulation Questionnaire (Gross and John, 2003). Such explorations could provide deeper insights into behavior regulation and more avenues for self-control intervention, suggesting a more nuanced approach to study self-control's role in achieving positive life outcomes.
